# Nanopore-Based, Real-Time Single-Molecule Probing of i‑Motif Structural Dynamics and Targeted PNA Disruption

**DOI:** 10.1021/acs.nanolett.5c06277

**Published:** 2026-02-02

**Authors:** Adina Cimpanu, Jonggwan Park, Loredana Mereuta, Yoonkyung Park, Tudor Luchian

**Affiliations:** † Department of Physics, Alexandru I. Cuza University, 700506 Iasi, Romania; ‡ Department of Bioinformatics, 65361 Kongju National University, Kongju 32588, Republic of Korea; § Department of Biomedical Science and Institute for Peptide Drugs (IPD), Chosun University, Gwangju 61452, Republic of Korea

**Keywords:** i-Motif, Peptide Nucleic Acid (PNA), Nanopore Sensing, pH-responsive Nanostructures

## Abstract

The i-motif, a crucial noncanonical DNA structure, is prevalent in gene regulatory regions, yet its dynamics is challenging to probe. Here, we employ a wild-type α-hemolysin nanopore (α-HL) to sense the folding of a human telomeric i-motif. We demonstrate two distinct sensing paradigms: reversible i-motif collisions at the nanopore’s β-barrel, producing transient current signatures, versus vestibule-first entry, yielding quasi-permanent blockades. The collision mode enables continuous i-motif dynamics monitoring, while vestibule entrapment provides ground for resolving pH-dependent volumetric changes in nanoconfinement with ∼nm^3^ resolution. We show that a short 6-mer peptide nucleic acid (PNA) complementary to the C-rich strand acts as a reversible antisense switch, capable of controllably invading and destabilizing the i-motif—an effect that is particularly pronounced when PNA binding precedes pH-induced folding. This work establishes a powerful single-molecule tool for investigating i-motif interactions and highlights new design principles for therapeutic PNAs by targeting i-motif-mediated regulatory structures.

## Graphical abstract

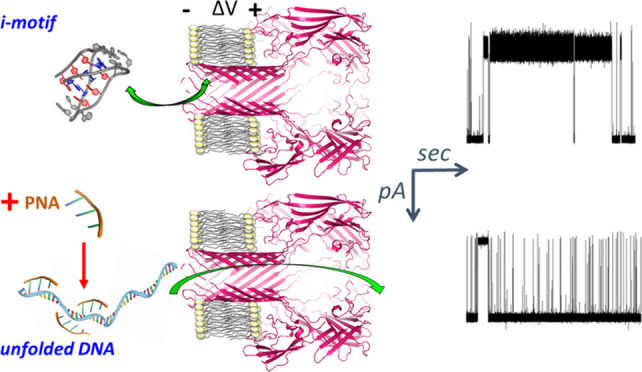

In addition to the B-form double helix molecular model of the DNA structure introduced 1953 by Watson and Crick,^1^ several other advanced structures were discovered including A-DNA, Z-DNA, triplex DNA, hairpin and cruciform structures, G quadruplexes (G4).^2^ In particular, helical structures known as i-motif^3^ are prevalent in genomic and there exists experimental evidence for the role of i-motif in various biological processes. Structurally, the i-motif consists of two parallel duplexes intercalated in an antiparallel orientation through hemiprotonated cytosine–cytosine base pairs especially stable in low pH environments, it can form from two or four DNA strands (intermolecular) or folds from a single strand (intramolecular). Interestingly, the presence of cations or molecular crowding promote of i-motif formation in the C-rich strand even at near neutral pH.^4^ It has been shown that i-motif forming sequences are common in the genome, and numerous studies have described their potential role in processes including gene transcription,^5^ DNA synthesis^6^ and pinpointed their presence in the promoter regions of oncogenes.^7^ As reported, various methods proved useful for i-motifs characterization, including: nuclear magnetic resonance (NMR),^8^ circular dichroism (CD) spectroscopy^9^ or fluorescence resonance energy transfer.^10^

Within the paradigm of targeting i-motifs to offer innovative pathways for cancer treatment, several small molecule ligands have been reported to interact with i-motifs and arguably play important roles in regulating the biological activities of specific genes.^11^ While the bulk methods have well contributed to the detection, functional and structural analysis of folded DNA structure, and in particular of i-motifs, there is an urgent need for improved approaches permitting real-time exploration of the stability, intermediate folding states, and kinetic evolution of i-motif nanoassemblies with high sensitivity, under naturally occurring environments where i-motif forming sequences experience topological constraints.

To this end, single-molecule level techniques offer unprecedented insights to detect, understand and explore the formation of these structures.^12,13^ Leveraging the high sensitivity, relatively low operational cost and ease of use, nanopores and in particular biological nanopores^14–16^ have been extensively used as a platform for label-free, single-molecule sensing of biomolecules including DNA,^14,17,18^ peptides,^19–22^ proteins,^23^ or other small molecules.^24,25^

With direct relevance to the present work, the α-HL nanopore facilitated single-molecule studies on the folding/unfolding kinetic properties of the G-quadruplexes^26,27^ and proved extremely successful to monitoring the pH dependent folded structures, stability and dynamics of the i-motif structure.^28–30^

This Letter reports the single-molecule study of a human telomere i-motif using an α-HL nanopore. First, we demonstrate i-motif detection and compare the electrophoretic capture of it from the β-barrel and vestibule entrances. Using pH titration, we leverage these distinct capture modes to monitor folding/unfolding dynamics in each configuration. Second, we explore the action of i-motif modulators, specifically a short PNA strand, whose mechanism is proposed to impose conformational constraints that rigidify the i-motif structure. As a landmark in our strategy, we implicated PNA^31^ which were already proposed as an attractive choice for numerous applications involving antisense agents, diagnostic probes, or modulation of gene expression.^32^ Our single-molecule analysis reveals that a short, nonfunctionalized 6-mer PNA targeting the 5′-CCC TAA-3′ cytosine tract can stabilize the DNA against low pH-induced i-motif formation.

For concreteness, we focused on the minimal human telomere single-stranded i-motif sequence, having a poly-2′-deoxyadenosine (dA_9_) extension on the 3′ end (5′-CCC TAA CCC TAA CCC TAA CCC AAA AAA AAA-3′) (denoted herein by DNA; Table S1), to assist threading of the i-motif inside the α-HL. The detailed methods and reagents used throughout are reported in the accompanying Supporting Information material.

As a preliminary test prior to exploring the single-molecule, real-time detection of pH-dependent folding of the proposed DNA, UV–vis difference spectra were measured^29^ to establish i-motif formation propensity in the low pH regime, under the high ionic strength implicated in nanopore experiments. In Figure S1 we display representative original and difference spectra for the DNA fragment revealing the progressive apparition of a minimum at ∼ 292 nm and a maximum at ∼ 240 nm with lowering the buffer pH, characteristic of i-motif folds. Having established this, we turned to the nanopore-based detection to investigate if and how the single-molecule signature of αHL-DNA blockade events correlate with the pH-dependent (un)­folded state of the DNA. We anticipated that if present, such events become visible in a reasonable time during experiments, as it was reported previously that the folding/unfolding of a similar DNA sequence (CCC TAA)_4_ occur on the time scale of ∼ subminutes at acidic pH.^13^

As a new approach, DNA fragments were added to the *trans* side of the nanopore and electrophoretically driven into the β-barrel nanocavity at negative transmembrane potentials (-ΔVs). To achieve an optimal acidic environment for i-motif formation and detect the ensuing folded DNA species during the same experiment, the electrolyte pH was modified by incremental addition of predetermined volumes from a stock solution of HCl, on the *trans* side of the nanopore only.

At a neutral pH = 6.96 and ΔV = −130 mV, the captured DNA molecules yield short downward spikes in the open nanopore current (Figure 1a). All-point histogram analysis showed a heterogeneous population of distinct blockades, suggestive of partly folded (low blockade B1 state) and respectively unfolded DNA (deep blockade B2 state) entering the nanopore’s β-barrel on the *trans* side (Figure 1a). For effectiveness, the nanopore blockade propensity was assessed herein through a lumped blockade probability, calculated as the histogram area associated with the presence of B1 and B2 events colored in orange in Figure 1, relative to the total histogram area. We noted that as we gradually increased the *trans* solution acidity, longer lasting B1 and B2 events emerged (Figure 1b,c), and this is consistent with our hypothesis that such acidic pH_*trans*_ values create optimal conditions for the formation of fully or partially folded i-motif DNA species, likely to plug for longer times the nanopore upon capture. This was due to the presence of the dA_9_ homopolymer tail assisted the threading of the folded DNA into the β-barrel, allowing the i-motif to interact and plug the α-HL, most likely impeding translocation. Further lowering of pH_*trans*_ to ∼ 3.86, produced blockades displaying only short-lived events (Figure 1, d) with a virtually absent fraction of longer-lived blockage current levels, that in turn resembled a similar appearance to the case of interactions recorded near neutral pH (Figure 1, a). This suggests transitions of the DNA fragments from i-motif to random coil at pH_*trans*_ ∼ 3.86 (see also the statistics on ionic current blockades illustrated in Figure 1, presented in Table S2. For example, at the extreme high and low trans pH values, namely pH_*trans*_ = 6.96 and 3.86, the residual currents I_B2_ are practically similar (I_B2; pH_*trans*_=6.96)_ = −24.5 ± 0.3 pA and I_B2; pH_*trans*_=3.86)_ = −25.7 ± 2.1), suggesting a similar topology of an (unfolded) DNA fragment blocking the protein’s β-barrel. However, at pH_*trans*_ ∼ 4.64 - which is close to the p*K*_a_ of free cytosine in bulk of ∼ 4.6, maximizing the number of C:C^+^ base-pairs in the i-motif – the B1 and B2 blocked states are convoluted, indicative of an i-motif fragment which cannot enter the nanopore’s β-barrel due to topological constrains. Similar experiments carried out at ΔV = −100 mV (Figure S2), establishes that monitoring the reversible α-HL-DNA interactions from the *trans* side, represent a convenient method for reporting in real time the low pH-induced i-motif emergence.

**Figure 1:**
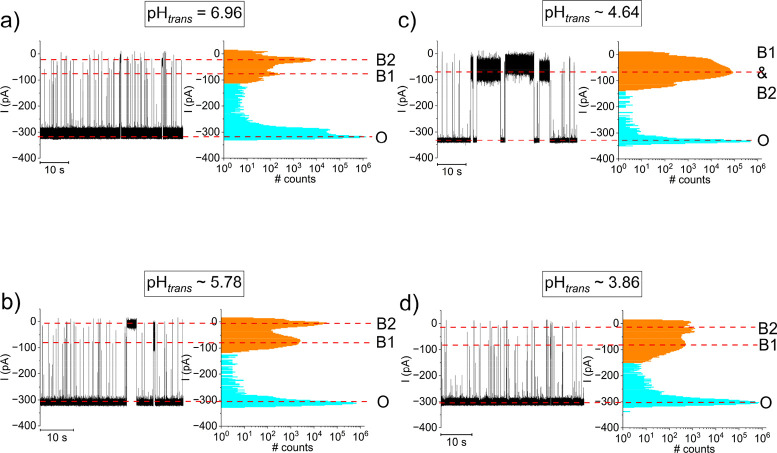
Detection of i-motif at the β-barrel entrance of α-HL. Representative, single-channel current traces of 2 μM *trans*-added DNA capture and transport through an α-HL, recorded at ΔV = −130 mV in symmetrical 3 M KCl buffered with 10 mM HEPES at pH 6.96 (a) and around pH_*trans*_ ∼5.78 (b), ∼4.64 (c) and ∼3.86 (d). Included for each representative trace are the corresponding all-point histograms, unraveling the ionic currents for the α-HL’s open state (O), and the commonly found blockade levels induced by DNA-α-HL interactions, denoted by B1 and B2.

As the exterior cross-sectional size of a similar i-motif (length of ∼ 3.1 nm and cross-sectional dimensions of ∼ 2 nm x 2 nm)^28^ exceeds the size of the most constricted region of α-HL (diameter of ∼ 1.4 nm), thus impeding translocation, we speculated that under the electrophoretic force, the i-motif would be pulled in an unfolded, single-stranded state across the constriction.

Instead, we find that the i-motif-characteristic blockade events seen around pH ∼ 4.64 increase in duration as ΔV increases (Figure 2, a, b) indicating a behavior whereby the captured DNA returns to bulk solution in the *trans* side, without unfolding and translocation.

**Figure 2:**
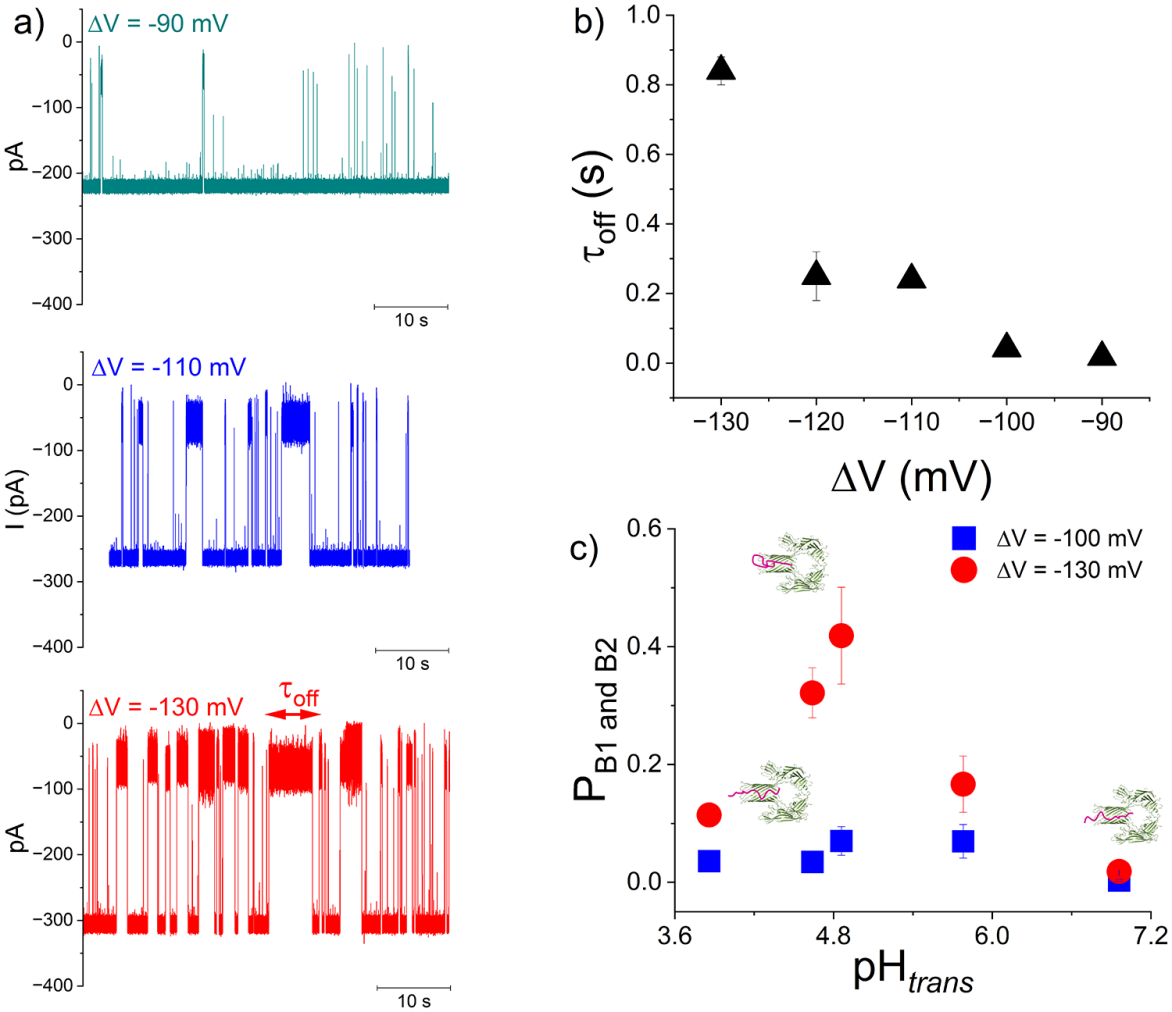
Nanopore analysis of i-motif forming propensity vs low pH. (a) Selected traces displaying the interaction of *trans*-added [2 μM] DNA with a negatively biased α-HL at the indicated ΔVs, measured at pH_*trans*_ ∼ 4.64. (b) The voltage dependence of the DNA-α-HL dissociation time (τ_off_) measured at pH_*trans*_ ∼ 4.64. (c) Quantitative analysis of the pH-dependent folding of *trans*-added DNA, indicative of i-motif formation, calculated in terms of occupancy probability of substates B1 and B2 of the negatively biased α-HL, through the histogram areas associated with the presence of B1 and B2 events relative to the total histogram areas showing in the corresponding all-point histograms (see also Figure 1). The sketches in (c) suggest unfolded DNA capture by α-HL at neutral and pH_*trans*_ ∼ 3.86, and respectively of i-motif DNA capture at pH_*trans*_ ∼ 4.64 and 4.86.

To help gauge the nanopore’s ability to report on how electrolyte acidity triggers i-motif formation, we plotted the pH-dependent blockade probability of the nanopore while interacting with a DNA fragment, via the lumped occupancy of B1 and B2 substates (P_B1 and B2_), at ΔV = −100 and −130 mV (Figure 2, c). Our results reveal a window of *trans* pH values (pH_*trans*_ ∼ 4.64 and 4.86) associated with the emergence of largest P_B1 and B2_ values. Knowing that an i-motif folds optimally in the pH range from 4.2 to 5.7, our findings suggest a scenario whereby at pH_*trans*_ ∼ 4.64 and 4.86 as used herein, DNA fragments transition from the single-stranded to folded i-motif conformations, which are precluded from passage through the nanopore; instead, they clog the β-barrel entrance for longer times, most likely assisted by the dA_9_ extension which threads the i-motif domain inside the α-HL, hence the larger P_B1 and B2_.

It should be noted that in previous work implicating a distinct ssDNA sequence (dA_20_) not amenable to i-motif formation, authors have revealed the appearance of deep blockades upon interaction with the α-HL nanopore with an increased event residence time in low pH conditions, also explained via the (low) pH effect on DNA secondary structure.^33^

Similar experiments as above were undertaken with the DNA fragments added from the *cis* side, whereby an electrophoretic force would determine their capture from the vestibule entrance of α-HL. The selected traces recorded at a pH_*cis*_ ∼ 4.64 - known to favor i-motif folding - revealed a set of very long and stable blockades on the open α-HL (Figure 3, a-d), unlike the population of reversible blockades recorded at pH = 6.96 (Figure 3, e). These longer lasting blockades persisted throughout the observation time in our experiments, and usually a reversal of the transmembrane potential was needed in order to restore the fully conductive state of the nanopore, as previously described.^28,30^

**Figure 3:**
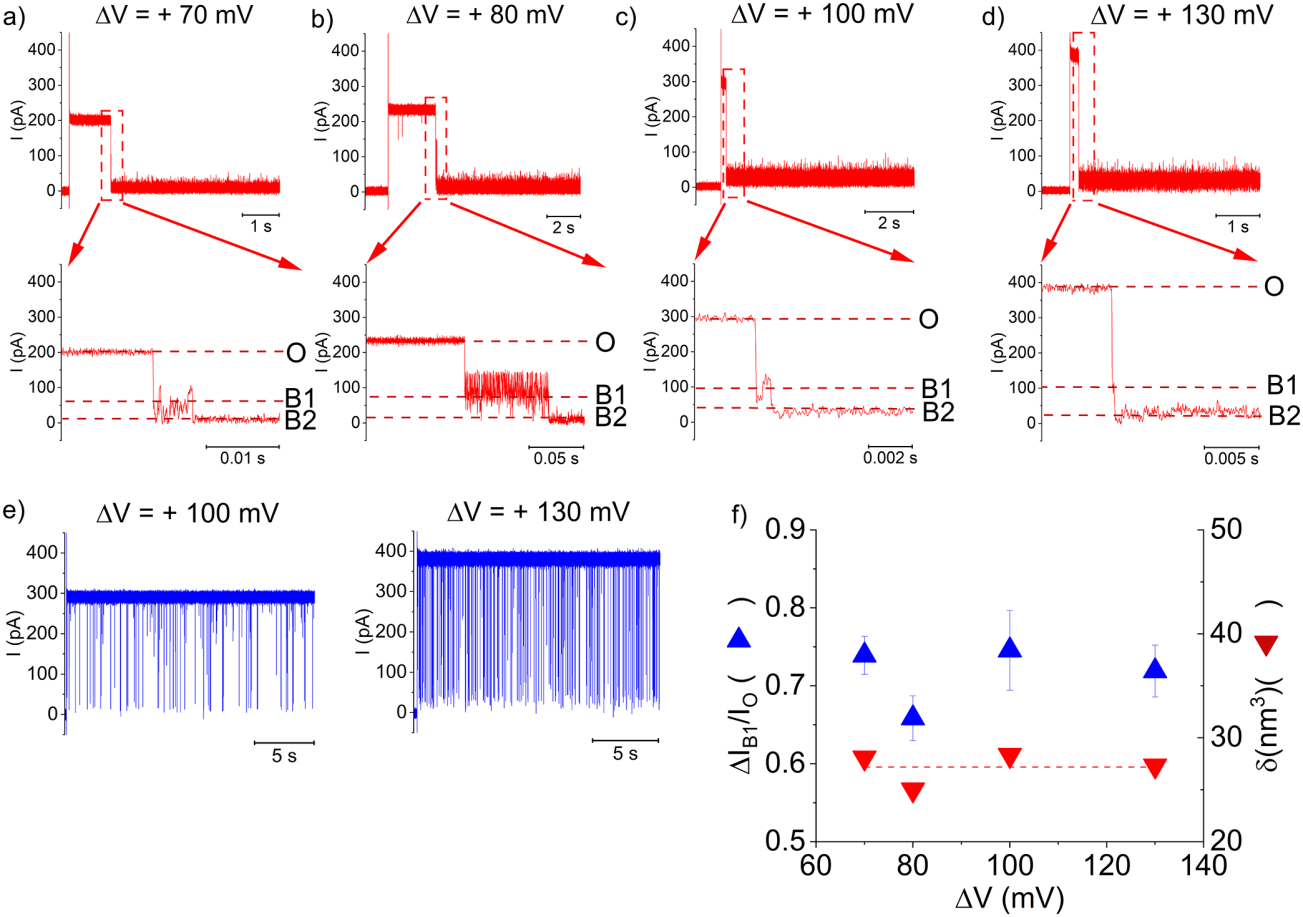
The irreversible capture of an i-motif inside α-HL’s vestibule presents unique opportunities for volumetric analysis in confined spaces. (a–d) Representative traces illustrating the irreversible capture of *cis*-added DNA [2 μM] with the *cis* electrolyte buffered at pH_*cis*_ ∼ 4.64, recorded at distinct ΔVs. The zoomed-in excerpts show representative blockade events triggered by the i-motif captured inside the nanopore, termed B1 and B2. In (e) we represent similar representative recordings, but carried out at neutral pH = 6.96 at two distinct ΔV’s, as displayed. (f) Voltage-dependence of the relative nanopore blockades ( $\frac{\Delta I_{B1}}{I_{O}}$ ) elicited by a vestibule-trapped DNA ((i-motif and the appended dA_9_ extension) and the corresponding, calculated molecular volumes of the DNA (δ). The dashed line represents the zero-slope linear fit of the δ vs ΔV plot.

Visual inspection revealed a two-step current blockade whose characteristic levels were termed B1 and B2 (Figure 3a–d, enlarged excerpts). B1 blockade events were interpreted as entry of the folded i-motif into the α-HL’s vestibule generating I_B1_, which subsequently transitioned practically irreversible to a deeper blockade state (B2) with a residual the current I_B2_. In this interpretation, the B2 blockade level was caused by a chain of events during which the single stranded moiety of the captured DNA enters the α-HL’s constriction region, pulled under the applied transmembrane potential. We also stress that the clear appearance of the consecutive blockade events O→B_1_→B_2_ is not ubiquitous to all recordings; occasionally, the captured DNA interacts very fast with the central constriction, rendering the B1 state absent.

The I_B1_ values were normalized to the I_O_, to report on the relative blockade extent while the i-motif was entrapped inside the α-HL’s vestibule, via $\frac{\Delta I_{B1}}{I_{O}}$ = $\frac{I_{B1-I_{O}}}{I_{O}}$ ­(Figure 3f), later used to estimate molecular volume of the i-motif. To simplify this analysis, the protein vestibule was viewed as a uniformly sized cylinder of length (l) and diameter (d), immersed in a buffer of electrical conductivity (σ), and clamped at a potential difference (ΔV). By neglecting the access resistance to the nanopore, the volume (δ) of a captured analyte viewed as a cylinder aligned parallel to the electric field inside the nanopore of volume (*v*_*vestibule*_) that entails a relative current blockage of ΔI can be estimated as before:^21^

$$\delta =\frac{\Delta I{\left(l\right)}^{2}}{\sigma \Delta V}=\frac{\Delta I_{B1}}{I_{O}}v_{vestibule}$$

Assuming an average value of d = 3.1 nm and l = 5 nm the α-HL’s vestibule diameter and respectively length, its volume was estimated at *v*_*vestibule*_ = 38 nm^3^. The volumetric analysis on data presented in Figure 3, f resulted in the volume (δ) of a captured DNA (i-motif and the appended dA_9_ extension) generated at pH_*cis*_ ∼ 4.64, δ ∼ 27.2 ± 0.7 nm^3^. For instructive comparison, a similar analysis of DNA-induced blockades seen from experiments undertaken a slightly less acidic (pH_*cis*_ ∼ 4.86), resulted in δ ∼ 19.8 ± 1.6 *nm* ^3^ (Figure S3).

We note that within the acidity domain in which the i-motif folds optimally, small pH changes (ΔpH_*cis*_ = 0.22) result in quite distinct values of the estimated volume for the vestibule-entrapped i-motif. We posit that at pH_*cis*_ ∼ 4.64, which is close to the p*K*_a_ of free cytosine in bulk (p*K*_a_ ∼ 4.6), a more rigid topology of the i-motif follows. By the same rationale, at pH_*cis*_ ∼ 4.86, the folded DNA is less stable, which reflects itself as a slightly less compact DNA topology, more prone to disorganization inside the α-HL’s vestibule, hence less voluminous. This assertion is supported by experiments carried out at pH_*cis*_ ∼ 5.78, whereby the estimated volume of the entrapped DNA—which is even less compact that at the previous acid pHs—was found δ ∼ 13.7 ± 1.1 nm^3^ (data not shown).

As demonstrated in Figure 2, we expected that if pH dropped below 4, a blockade pattern indicative of i-motif formation would be absent, as most of the cytosine bases become protonated. Quite surprisingly, the outcome of our experiments indicates a different result, namely: (i) at a pH_*cis*_ ∼ 3.86, the DNA trapped inside the α-HL’s vestibule still resembled an i-motif, judged through the ensuing very stable blockades and slow dissociation kinetics (Figure S4, I, a, b); (ii) only by further lowering the pH_*cis*_ to ∼3.68, the entrapped DNA was capable to exhibit reversible blockades indicative of unfolded DNA appearance (Figure S4, II, a, b). The full statistics on the blockade currents is reported in Table S3.

A reasonable cause for this unexpected observation is that the confined nanocavity of the α-HL vestibule stabilizes the i-motif structure, as the i-motif→coil transition may require greater energy to unfold in such a crowded environment vs bulk solution.^34^

An additional objective of this work was to discover new pathways to influence the propensity of the chosen nucleotide sequence to follow a transitional pH for i-motif formation. To this end, we resorted to hybridization-based targeting employing short 6-mers PNA sequences, complementary to the repeats of the cytosine tract (5′-CCC TAA-3′) critical to the stability of the i-motif, and tested their capability of interfering with i-motif formation at acidic pH ∼ 4.64. The feasibility of this approach is demonstrated in part by previous research demonstrating that short PNA sequences in the form of 5- or 7-mers do hybridize with target polynucleotide sequences.^35^

As an additional control carried out herein, we illustrate that when both 6-mers PNA and DNA fragments are mixed on the *trans* side of the nanopore near neutral pH, the stochastic blockade events elicited are suggestive of capturing DNA-PNA complexes (Figure S5). This conclusion is also strengthened by previous findings regarding the DNA-PNA complexes a microscopic association binding constant (∼14 M^–1^ bp^–1^),^36^ corresponding to a standard Gibbs free energy ∼ −40 kJ mol^–1^ at room temperature (298 K) for relatively stable 6-mers duplexes.

We investigated two distinct scenarios in which probe PNA fragments targeted the DNA sequences, namely before and after acidic pH changes intended to trigger i-motif formation. The rationale was to establish whether the low pH-triggered, i-motif stabilizing C:C^+^ hydrogen bonding competes with the formation of DNA-PNA duplexes, as in first scenario, or DNA-PNA interactions are fully capable of reversing i-motif folding, as in the second scenario.

To provide a benchmark for the DNA-PNA molar ratio expected to generate a significant binding, we assumed a simple bimolecular interaction between the DNA target and PNA ligand, whereby the tight DNA-PNA binding depletes the free PNA concentration. In this framework, the equilibrium concentration of the DNA-PNA complex is given by [*PNA* – *DNA*] = $\frac{\left(3\left[DNA\right]+\left[PNA\right]+K_{d}\right)-\sqrt{{\left(3\left[DNA\right]+\left[PNA\right]+K_{d}\right)}^{2}-4\left[PNA\right]3\left[DNA\right]}}{2}$ , where [DNA], [PNA] and *K*_*d*_ indicate the aqueous concentration of DNA, PNA and respectively 6-mer DNA-PNA duplex dissociation constant (1.3 × 10^–7^ M).^36^ By virtue of multivalency, i.e. each DNA sequence has three equivalent binding sites (5′-CCC TAA-3′) for the complementary PNA, the actual molarity of DNA in the formula above was multiplied by 3. Considering that binding of a single PNA at any of the three available sites on the DNA fragment suffices to interfere with the i-motif folding as well as the lack of cooperativity, simple calculations indicate that when micromolar concentrations of DNA and PNA are being mixed at a molar ratio of approximately 1:1.5, a half value of [*DNA* – *PNA*] relative to its saturation plateau ensues. Hence, to maximize the expected effect, in our experiments we employed larger than 1:1.5 DNA-PNA molar ratios.

The representative traces in Figure 4 illustrate that with *trans* added DNA and subsequent to i-motif formation at pH_*trans*_ ∼ 4.64 (Figure 4, I, II, b), excess addition of the 6-mers PNA does not destabilize the i-motif (Figure 4, I, II, c), which would otherwise be seen as the emergence of short blockade events suggestive of unfolded DNA passage across α-HL (Figure 4, I, II, a). This is also judged through the estimating the occupancy probability of B1 and B2 states (see also Figure 2): at ΔV = −100 mV and with pH_*trans*_ ∼ 4.64, the P_B1 and B2_ probability equals P_B1 and B2_ = 0.06 ± 0.01 in the absence of PNA, and P_B1 and B2_ = 0.08 ± 0.03 with PNA present at the specific molar ratio. Similarly, at ΔV = −130 mV and with pH_*trans*_ ∼ 4.64, the P_B1 and B2_ probability equals P_B1 and B2_ = 0.2 ± 0.1 in the absence of P_B1 and B2_ = 0.5 ± 0.07 with PNA present in solution. A similar phenomenon was observed when experiments were carried out with the *cis*-side added DNA (Figure S6 and Table S4).

**Figure 4:**
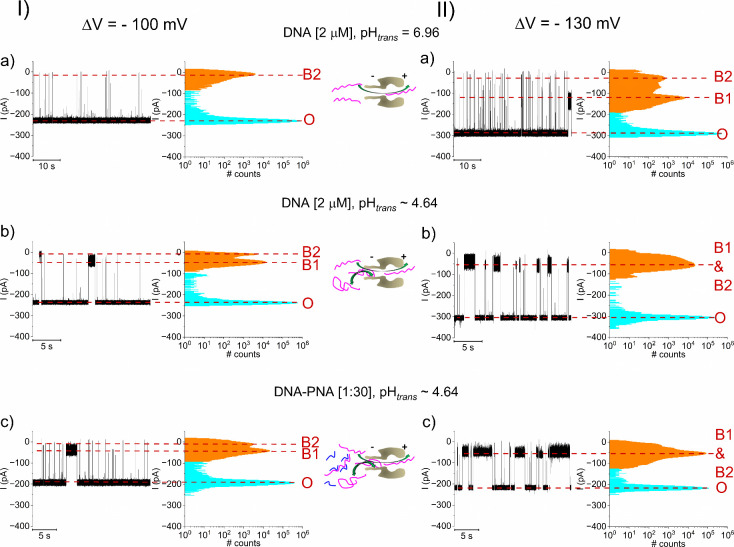
DNA-PNA interactions fail to interfer with the low pH induced i-motif formation if acidity change preceds 6-mers PNA addition. Real-time, single-molecule monitoring of stochastic events illustrative of *trans*-added DNA-α-HL reversible interactions at neutral pH and respectivley pH_*trans*_ ∼ 4.64 recorded at ΔV = −100 mV (I, a, b) and ΔV = −130 mV (II, a, b). The i-motif emergence at acidic pH is indicated by the prevalence of blockade substates B1 and B2 (vide supra). Subsequent addition of 6-mers complementary PNA on the *trans* side, to achieve a DNA-PNA molar ratio of 1:30, leaves the i-motif appearance largely invariant at the applied voltages of ΔV = −100 mV (I, c) and respectively ΔV = −130 mV (II, c). The shown sketches illustrate DNA capture by α-HL at neutral (I, II, a) and pH_*trans*_ ∼ 4.64 (I, II, b), and respectively of DNA­(magenta)-PNA­(blue) fragments pH_*trans*_ ∼ 4.64 (I, II, c).

In stark contrast, we discovered that the low pH-induced i-motif formation could be blocked via PNA-DNA hybridization, when the *trans*-added fragments are allowed to interact at neutral pH *before lowering the pH* in the working electrolyte. In Figure 5, I, II, a, we represent the reversible current blockades entailed by *trans*-added DNA upon capture at the β-barrel entrance and passage across the negatively biased α-HL. As shown, mixing target DNA and probe 6-mers PNA at neutral pH_*trans*_ (molar ratio [1:30], followed by the subsequent capture of formed complexes at the β-barrel opening, result in blockades presented in Figure 5, I, II, b, which show little variation in terms of amplitudes, even upon lowering the pH_*trans*_ to ∼ 4.64 (Figure 5, I, II, c) (see Table S5). This argues in support of a rather similar topological nature of DNA fragments eliciting the stochastic events in Figure 5, I, II, b, c. In terms of occupancy probabilities, at ΔV = −100 mV, DNA-PNA [1:30] and neutral pH (Figure 5, I, b) P_B1 and B2_ = 0.004 ± 0.001 whereas at pH_*trans*_ ∼ 4.64 (Figure 5, I, c), P_B1 and B2_ = 0.0033 ± 0.001. Similarly, at ΔV = −130 mV, DNA-PNA molar ratio of 1:30 and neutral pH (Figure 5, II, b), P_B1 and B2_ = 0.022 ± 0.003 whereas at pH_*trans*_ ∼ 4.64 (Figure 5, II, c) P_B1 and B2_ = 0.026 ± 0.01. Control experiments demonstrated that dimethyl sulfoxide (DMSO) added at neutral pH and concentration almost double (∼ 15%) than the largest obtained upon PNA pipetting, did not preclude i-motif formation upon subsequent changing the pH to ∼ 4.64 (Figure S7). This indicates the lack of DMSO interference with the DNA folding in low pH.

**Figure 5:**
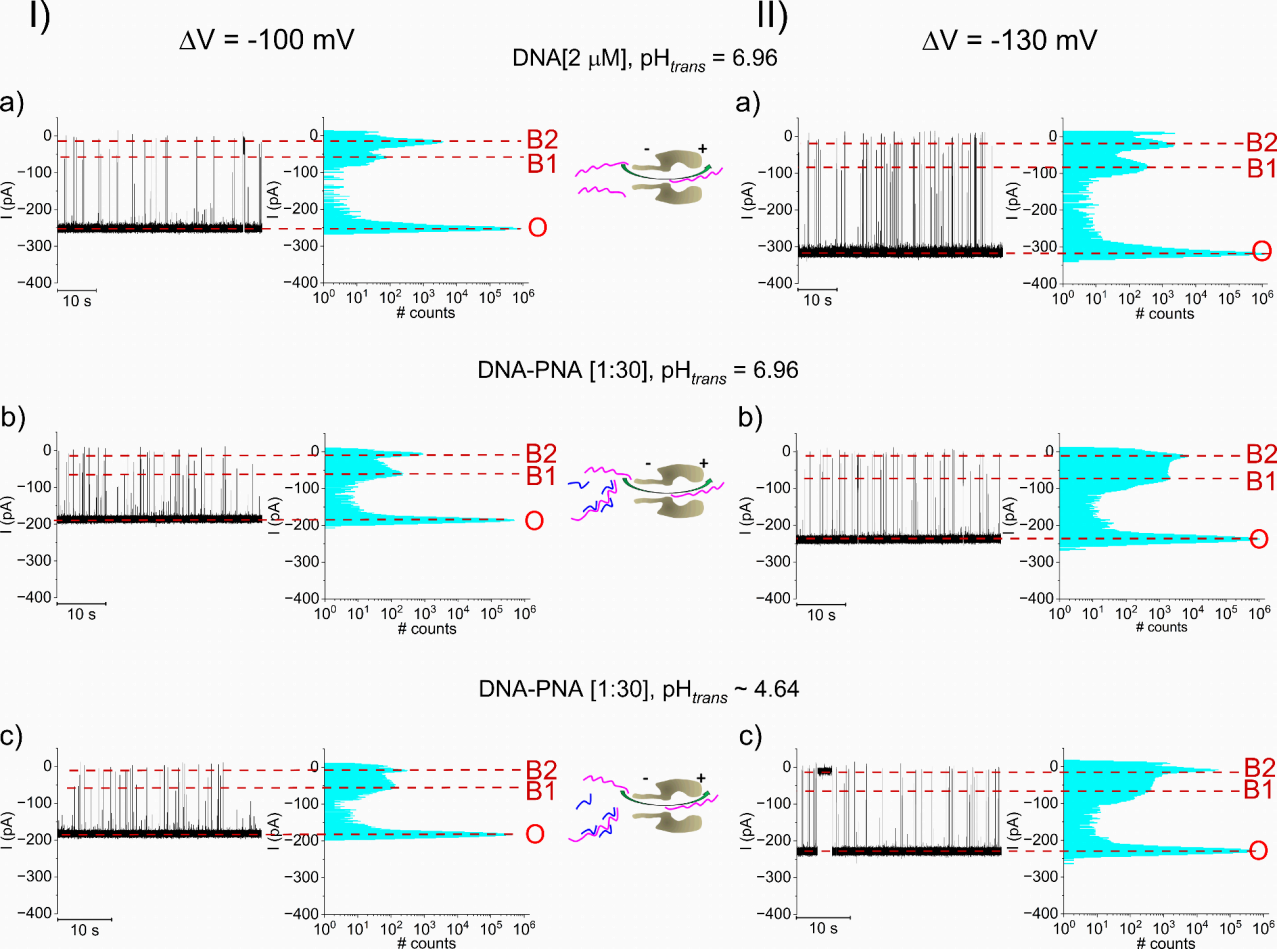
DNA-PNA interactions preclude the low pH-induced i-motif formation if 6-mers PNA addition precedes acidity changes. Selected traces displaying the blockade events elicited by *trans*-added DNA-α-HL reversible interactions around neutral pH = 6.96 at ΔV = −100 mV (I, a) and ΔV = −130 mV (II, a). Next, we display representative blockade events elicited across α-HL recorded at ΔV = −100 mV (I, b) and ΔV = −130 mV (II, b), but following the *trans* side, 6-mers PNA addition at a DNA-PNA molar ratio of [1:30], also at pH = 6.96. Subsequent acidity changes to pH_*trans*_ ∼ 4.64 does not alter the blockage pattern of DNA-PNA-α-HL interactions, indicating the lack of low pH-induced i-motif formation on the DNA fragments, which would otherwise appear as in Figure 4, I, II, c. The shown sketches illustrate DNA (I, II, a) and DNA­(magenta)-PNA­(blue) fragments capture by α-HL at neutral pH (I, II, b), and respectively of DNA­(magenta)-PNA­(blue) fragments pH_*trans*_ ∼ 4.64 (I, II, c).

Further support for this finding came from experiments whereby DNA-PNA fragments preincubated at neutral pH, at different molar ratios (1:20, 1:30 and 1:40), were captured from the *cis* side of α-HL, at pH_*cis*_ ∼ 4.64. Figure S8 reveals that with increasing molar contributions of PNA to the DNA-PNA mixture, multiple reversible blockade events become visible, resulting from the transient capture of DNA-PNA fragments on the vestibule, followed by passage across nanopore. The arguments for this are 2-fold: (i) the statistical analysis of the event lifetimes characterizing the fragments dissociation (τ_off_) from the nanopore – descriptive of the B2 substate - recorded at ΔV = +100 mV and pH_*cis*_ ∼ 4.64, at the [DNA-PNA] molar ratios where such occurrences are more prevalent (Figure S8 and Table S6), revealed values of τ_off_ (s) = 11 × 10^–3^ ± 2.3 × 10^–4^ (average ± SEM) (DNA-PNA [1:30]) and respectively τ_off_ (s) = 4.2 × 10^–4^ ± 0.11 × 10^–4^ (average ± SEM) (DNA-PNA [1:40]). Although such values are larger than those characterizing free DNA dissociation (τ_off_) from the nanopore around neutral pH (Figure 3, e, ΔV = +100 mV) (τ_off_ (s) = 4.7 × 10^–5^ ± 2.4 × 10^–4^ (average ± SEM)) (see also Figure S5 for data on translocation time of DNA-PNA fragments), in previous work we demonstrated that the pH-dependent ionization of amino acids lining the nanopore’s translocation pathway can generate a slowed down passage of DNA fragments, at pH ∼ 4.7;^37^ (ii) the B2 level relative blockades measured at ΔV = +100 mV, around neutral pH (free DNA only) and at pH_*cis*_ ∼ 4.64 (various [DNA-PNA] molar ratios) (Figure S8 and Table S6), were found close: $\frac{\Delta I_{block}}{I_{O}}$ = 0.91 ± 0.004 (free DNA, pH = 6.94), $\frac{\Delta I_{block}}{I_{O}}$ = 0.8 ± 0.01 (DNA-PNA [1:30], pH_*cis*_ ∼ 4.64) and respectively $\frac{\Delta I_{block}}{I_{O}}$ = 0.84 ± 0.01­(DNA-PNA [1:40], pH_*cis*_ ∼ 4.64) (average ± SEM).

These support the finding that DNA-PNA hybridization at neutral pH stabilize DNA fragments into a rigid conformation, impeding subsequent C:C^+^ interactions that would promote i-motifs formation in low pH conditions. Gratifyingly, such findings are in excellent agreement with previous research, which established that short PNAs can perform as i-motif^38^ or G-quadruplex ligands,^35,39^ by forming strings of short duplexes, and the DNA-PNA binding requires lack of the pre-existent G quadruplex.^39^

Data shown in Figure S8 also reveal that eventually, an irreversible occlusion of the nanopore takes place, leaving a blockade fingerprint similar to that recorded with DNA molecules added alone in the electrolyte, in low pH_*cis*_ ∼ 4.64, as in Figure S6, I, II, a. This can be explained arguing that under equilibrium conditions, at the largest DNA-PNA molar ratio tested, there may still be a fraction of PNA-free DNA fragments, rendering them prone to undergoing i-motif folding around pH_*cis*_ ∼ 4.64.

In summary, we comprehensively explored the use of the α-HL nanopore to detect and characterize a model i-motif DNA sequence at the single-molecule level, probing its structural dynamics, pH sensitivity, and interactions with short PNA ligands. Capture and entrapment of the i-motif within the α-HL vestibule produced an irreversible characteristic current blockade. In contrast, electrophoretic driving of the i-motif toward the β-barrel entrance resulted only in brief, reversible blockades due to collisions that prevented full translocation. Crucially, the two paradigms offer distinct analytical capabilities, namely: (i) DNA capture inside the α-HL’s vestibule facilitates estimation and discrimination of the i-motif volume under nanoconfinement; (ii) DNA capture at the β-barrel enables the real-time, continuous detection of folded i-motif sequences, including cases where folding is coupled to ligand binding.

We demonstrate that a 6-mer PNA—designed to hybridize with the cytosine-rich tract (5′-CCC TAA-3′)—can compete with i-motif formation. This PNA effectively stabilizes the DNA against low pH-induced folding, provided it binds before the pH is lowered. Considering the intrinsic advantages of PNAs, e.g. greater resistance to degradation by protease or nuclease, we envision potentially lucrative extensions of the presented approach, either by coupling probe PNAs with cell-penetrating peptide (CPP) to assist exogenous delivery of such synthetic hybridization probes into cells, or adding flanking nucleotide sequences to the probe PNAs, to compensate for the target secondary structure and favor i-motif invasion. We anticipate that in doing so, the presented approach has the potential of translational applicability in PNA-based therapeutics, aimed at blocking transcription of genes containing i-motif promoter sequences.

## Supplementary Material



## Notes

Additional experimental details, materials, and methods, including tables and supplementary figures (PDF)
