# Supplementary material for: Nanopore-Based, Real-Time Single-Molecule Probing of i‑Motif Structural Dynamics and Targeted PNA Disruption
Source: Nano Lett. 2026 Feb 2;26(6):2316–24. doi: 10.1021/acs.nanolett.5c06277 (PMC12958338; doi:10.1021/acs.nanolett.5c06277)
Supplement: Supplementary file 1 [file nl5c06277_si_001.pdf]

Supporting Information

**Nanopore-Based, Real-Time Single-Molecule Probing of i-Motif Structural Dynamics and Targeted PNA Disruption**

Adina Cimpanu[1], Jonggwan Park[2], Loredana Mereuta[1,*], Yoonkyung Park[3,*], Tudor Luchian[1,*]

[1]Department of Physics, Alexandru I. Cuza University, 700506 Iasi, Romania

[2]Department of Bioinformatics, Kongju National University, Kongju 32588, Republic of Korea,

[3]Department of Biomedical Science and Institute for Peptide Drugs (IPD), Chosun University, Gwangju 61452, Republic of Korea.

[*]Corresponding authors: loredana.mereuta@uaic.ro, y_k_park@chosun.ac.kr, luchian@uaic.ro

## MATERIALS AND METHOD

**Reagents.** Potassium chloride (KCl), dimethyl sulfoxide (DMSO), ultra-pure water (DNAase and RNAase free), HEPES (N-(2-Hydroxyethyl)piperazine-N′-(2-ethanesulfonic acid) buffer, *n*-pentane HPLC-grade (10 mg/mL), hexadecane and wild-type α-hemolysin (α-HL) monomeric protein, were purchased from Sigma–Aldrich, Germany. The 1,2-diphytanoyl-sn-glycerophosphocholine (DPhPC) lipids were obtained from Avanti Polar Lipids, Alabaster, AL, USA. The PNA and i-motif DNA fragments were synthesized and purified by HLB Panagene Inc., Republic of Korea (PNA), according to the sequences designed by us. The primary sequences and molecular weights of the DNA and PNA are shown in Table S1.

**Samples preparation.** The dried DNA samples were dissolved in 1 M NaCl solution in ultra-pure water buffered with TE (10 mM Tris, 1 mM EDTA) at pH = 8.2, and vigorously stirred using a Stuart BioCote vortex mixer (Sigma–Aldrich, Germany) at 1,400 rpm, for 3 min to achieve a stock with a concentration of 200 μM. After solvation, the stock solution was heated up to 95 °C using an IKA Digital Block Heater (Cole-Parmer, USA) and slowly cooled down to 23 °C, to assure rehydration. A stock solution of PNA was obtained by hydrating the dry samples in the same buffer (TE) to a concentration of 278 μM, and DMSO was added to facilitate solvation. All liquid samples were vigorously stirred using a Stuart vortex-mixer with BioCote (Sigma−Aldrich, Germany) at 1400 rpm for 3 min each, then heated up to 95 °C for 20 min to improve rehydration. Aliquots of solution to be used in electrophysiology experiments were transferred into new vials and the remaining stock solutions were stored at −20 °C until further use. Prior to use in electrophysiology experiments, aliquots were heated to 95 °C using an IKA Digital Block Heater (Cole-Parmer, US) and slowly cooled down to 22 °C.

**Spectral analysis of pH-dependent i-motif formation.** To investigate the ability of DNA fragments to fold into i-motifs in low pH electrolyte, UV–vis spectroscopy experiments were performed using a NanoDrop OneC spectrophotometer (Thermo Fisher Scientific, USA). In such experiments, 1 mL of a reference solution containing 3 M KCl buffered with 10 mM HEPES around pH = 7 was pipetted in a quartz cuvette with a 10-mm path length, and the absorption spectra were recorded in the 190–850 nm wavelength range at a room temperature of ~ 22$^{o}$ C. Subsequently, an appropriate volume from a DNA stock solution (200 μM) were added, to attain a final concentration of 0.5 μM DNA in the cuvette. Absorption spectra were recorded in the similar wavelength range around neutral pH and upon lowering incrementally the pH in the buffer to different values, by adding pre-determined volumes from a HCl stock solution, checked via pH titration measurements for the ability to set the acidity as desired. Then, we simply took the arithmetic difference between the UV-vis spectrum in the unfolded state around neutral pH and the folded state at the various pH values set in the quartz cuvette with DNA present, to construct UV-vis difference spectra. To assess i-motif formation, we sought the characteristic emergence of a minimum at ~292 nm and a maximum at ~240 nm. Further, to qualitatively monitor the pH-dependent i-motif formation, we plotted the value of the minimum measured at $\lambda$ = 292 nm in the corresponding UV-vis difference spectra.

**Nanopore electrophysiology.** The bilayer chambers were separated by a 25 μm-thick Teflon film (Goodfellow, Malvern, MA, USA), containing an aperture of about 80 μm in diameter, across on which the lipid bilayer was obtained after pre-treated the film with a mixture of 1:10 hexadecane in n-pentane. Alternatively, and with even greater success, we used the manufactured Teflon septum with an aperture of 80 μm in diameter and Montal-Muller bilayer chamber from ELEMENTS SRL, Italy. Addition from a monomeric stock solution of ~ 0.5 to 8 μL of the wild-

type α-HL, to the grounded, *cis*-compartment under continuous stirring, led to insertion of a single heptameric nanopore into the formed stable lipid membrane. To attain pH changes from neutral to acidic values as reported herein, during the same experiment, HCl was added asymmetrically in a dropwise manner from a stock solution to either *cis* of *trans* chamber of the setup. Prior to this, we validated the right volume needed attain a given pH change in the electrolyte containing 3 M KCl buffered with 10 mM HEPES at pH = 6.96, by careful titration of the solution with known HCl volumes and subsequent recording of ensuing pH changes. All the measurements were carried out on a vibration-free platform (BenchMate 2210, Warner Instruments, USA), shielded in a Faraday cage (Warner Instruments, USA), at a room temperature of ~23°C. Ionic currents were recorded with an Axopatch 200B or Multiclamp 700B amplifier (Molecular Devices, USA) in the voltage-clamp mode and the electrical signals were digitized at a sampling frequency of 50 kHz with a NI PCI 6221 16-bit acquisition board (National Instruments, USA) and low-pass filtered at 10 kHz or 12 kHz. Alternatively, we employed for data recording and analysis the ePatch amplifier (ELEMENTS SRL, Italy). The numerical analysis of the ionic current blockades across the nanopore was performed within the statistics of exponentially distributed events using pClamp 6.03 (Axon Instruments, USA). Depending upon the experimental conditions and measurement constraints, the number of events used in histogram lifetime analysis ranged between 50 ÷ 280. All data reported herein and statistics stems from 3 ÷ 5 independent experiments.

**Molecular representation of the wild-type α-HL.** The molecular file of the WT α-HL was imported from RCSB Protein Data Bank (7AHL) into PyMOL ver. 2.5.7 (The PyMOL Molecular Graphics System, Version 2.0 Schrödinger, LLC.) ('PDB' file format) and resulting representations were later exported in graphic formats for subsequent reporting.

Table S1. Primary sequence of the i-motif-forming DNA and complementary 6-mers PNA fragments

| Fragment | Primary sequence | MW (g mole$^{-1}$) |
|---|---|---|
| DNA | 5'-CCC TAA CCC TAA CCC TAA CCC AAA AAA AAA (purity ≥ 90 %) | 9,019.18 |
| PNA | N-TTA GGG (purity ≥ 99.9 %) | 1698.6 |

Table S2. Statistics of the ionic currents mediated by the open α-HL nanopore ($I_O$) and of blockades induced following reversible captures of *trans*-added DNA describing substates B1 ($I_{B1}$) and respectively B2 ($I_{B2}$), measured at a transmembrane potential ΔV = -130 mV, and various $pH_{trans}$ values, as indicated also in Fig. 1.

| | $I_O$ (average ±SEM) | $I_{B1}$ (average ±SEM) | $I_{B2}$ (average ±SEM) |
|---|---|---|---|
| $pH_{cis}$ = 6.96<br>$pH_{trans}$ = 6.96 | -317.2 ± 0.04 pA | -77.6 ± 0.4 pA | -24.5 ± 0.3 pA |
| $pH_{cis}$ = 6.96<br>$pH_{trans}$ = 5.78 | - 310.6 ± 0.0 pA | - 74.1 ± 3.7 pA | - 6.04 ± 0.1 pA |
| $pH_{cis}$ = 6.96<br>$pH_{trans}$ = 4.86 | - 311.1 ± 0.01 pA | - 64.8 ± 0.14 pA | 0.7 ± 0.3 pA |
| $pH_{cis}$ = 6.96<br>$pH_{trans}$ = 4.64 | - 333.3 ± 0.02 pA | ($I_{B1\ and\ B2}$) - 62.7 ± 0.4 pA | |
| $pH_{cis}$ = 6.96<br>$pH_{trans}$ = 3.86 | -302.2 ± 0.1 pA | -74.2 ± 0.7 pA | -25.7 ± 2.1 pA |

Table S3. Statistics of the ionic currents mediated by the open α-HL nanopore ($I_O$), those accompanying blockades induced by vestibule-captured *cis*-added DNA describing the substate B2 ($I_{B2}$), and respectively the calculated relative blockade values of $I_{B2}$ measured at the indicated transmembrane potentials and pH values (see also Fig. S4).

| | ΔV (mV) | $I_O$ (average ±SEM) | $I_{B2}$ (average ±SEM) | $\frac{\Delta I_{B2}}{I_O}$ |
|---|---|---|---|---|
| pH = 6.96 trans<br>pH = 3.86 cis | + 100 | 297.2 ± 0.3 pA | 18.8 ± 0.1 pA | 0.94 |
| | + 130 | 401.3 ± 0.6 pA | 29.5 ± 0.3 pA | 0.93 |
| pH = 6.96 trans<br>pH = 3.68 cis | + 100 | 287.04 ± 0.07 pA | 20.7 ± 0.4 pA | 0.93 |
| | + 130 | 389.3 ± 0.06 pA | 17.5 ± 0.4 pA | 0.95 |

Table S4. Statistics of the ionic currents mediated by the open α-HL nanopore ($I_O$) and of irreversible blockades ($I_{B1}$ and $I_{B2}$) induced following irreversible *cis* capture of i-motif DNA in the absence and presence of 6-mers PNA at distinct molar ratios, at $pH_{cis}$ ~ 4.64, as shown also in Fig. S6.

| | **ΔV (mV)** | **$I_O$ (average ±SEM)** | **$I_{B1}$ (average ±SEM)** | **$I_{B2}$ (average ±SEM)** |
|---|---|---|---|---|
| **2 µM DNA cis** | + 100 | 288.9 ± 0.03 pA | largely absent | 29.9 ± 0.1 pA |
| | + 130 | 378.3 ± 0.06 pA | largely absent | 37.4 ± 0.1 pA |
| **DNA-PNA [1:5]** | + 100 | 256.2 ± 0.09 pA | 109.7 ± 1.1 pA | 21.1 ± 0.2 pA |
| | + 130 | 345.2 ± 0.1 pA | largely absent | 30.1 ± 0.4 pA |
| **DNA-PNA [1:20]** | + 100 | 253.1 ± 0.1 pA | 76.8 ± 0.8 pA | 29.7 ± 0.3 pA |
| | + 130 | 375.2 ± 0.06 pA | largely absent | 43.3 ± 0.5 pA |

Table S5. Statistics of the ionic currents mediated by the open α-HL nanopore ($I_O$) and of blockades induced following reversible captures of *trans*-added DNA describing substates B1 ($I_{B1}$) and respectively B2 ($I_{B2}$), measured at the shown transmembrane potentials, in absence or presence of PNA at distinct molar ratios and acidity values, as indicated also in Fig. 5.

| | **ΔV (mV)** | **$I_O$ (average ±SEM)** | **$I_{B1}$ (average ±SEM)** | **$I_{B2}$ (average ±SEM)** |
|---|---|---|---|---|
| **DNA**<br>**pH = 6.96 trans**<br>**pH = 6.96 cis** | -100 | -252.4 ± 0.01 pA | -59.1 ± 5.9 pA | -17.2 ± 0.07 pA |
| | -130 | -317.9 ± 0.02 pA | -81.8 ± 1.5 pA | -22.4 ± 0.2 pA |
| **DNA-PNA [1:30]**<br>**pH = 6.96 trans**<br>**pH = 6.96 cis** | - 100 | -186.3 ± 0.002 pA | -58.4 ± 7.2 pA | -6.1 ± 1.0 pA |
| | - 130 | -237.4 ± 0.006 pA | -47.5 ± 1.4 pA | -12.3 ± 0.2 pA |
| **DNA-PNA [1:30]**<br>**pH = 4.64 trans**<br>**pH = 6.96 cis** | - 100 | -182.9 ± 0.004 pA | -46.02 ± 0.8 pA | -8.8 ± 0.2 pA |
| | - 130 | -228.9 ± 0.007 pA | -55.4 ± 1.4 pA | -9.001 ± 1.3 pA |

Table S6. Statistics of the ionic currents mediated by the open α-HL nanopore ($I_O$) and of blockades induced following of *cis*-addition of a DNA-PNA mixture incubated at the indicated molar ratios at neutral pH, describing substates B1 ($I_{B1}$) and respectively B2 ($I_{B2}$), when the electrophysiology experiments were undertaken at $\Delta V = +100$ mV and $pH_{cis} \sim 4.64$, as indicated also in Fig. S8.

| | **$I_O$ (average ±SEM)** | **$I_{B1}$ (average ±SEM)** | **$I_{B2}$ (average ±SEM)** | **$\tau_{off}$ (s) (B2)** |
|---|---|---|---|---|
| **DNA-PNA [1:5]** | 286.8 ± 0.4 pA | largely absent | 22.1 ± 0.2 pA | irreversible block |
| **DNA-PNA [1:20]** | 288.4 ± 0.03 pA | 96.1 ± 0.7 pA | 21.1 ± 0.1 pA | $3.4 \times 10^{-3} \pm 2.3 \times 10^{-4}$ |
| **DNA-PNA [1:30]** | 282.5 ± 0.02 pA | largely absent | 31.8 ± 0.3 pA | $11 \times 10^{-3} \pm 2.4 \times 10^{-4}$ |
| **DNA-PNA [1:40]** | 251.6 ± 0.1 pA | 91.7 ± 4.8 pA | 14.1 ± 0.2 pA | $0.4 \times 10^{-3} \pm 0.1 \times 10^{-4}$ |

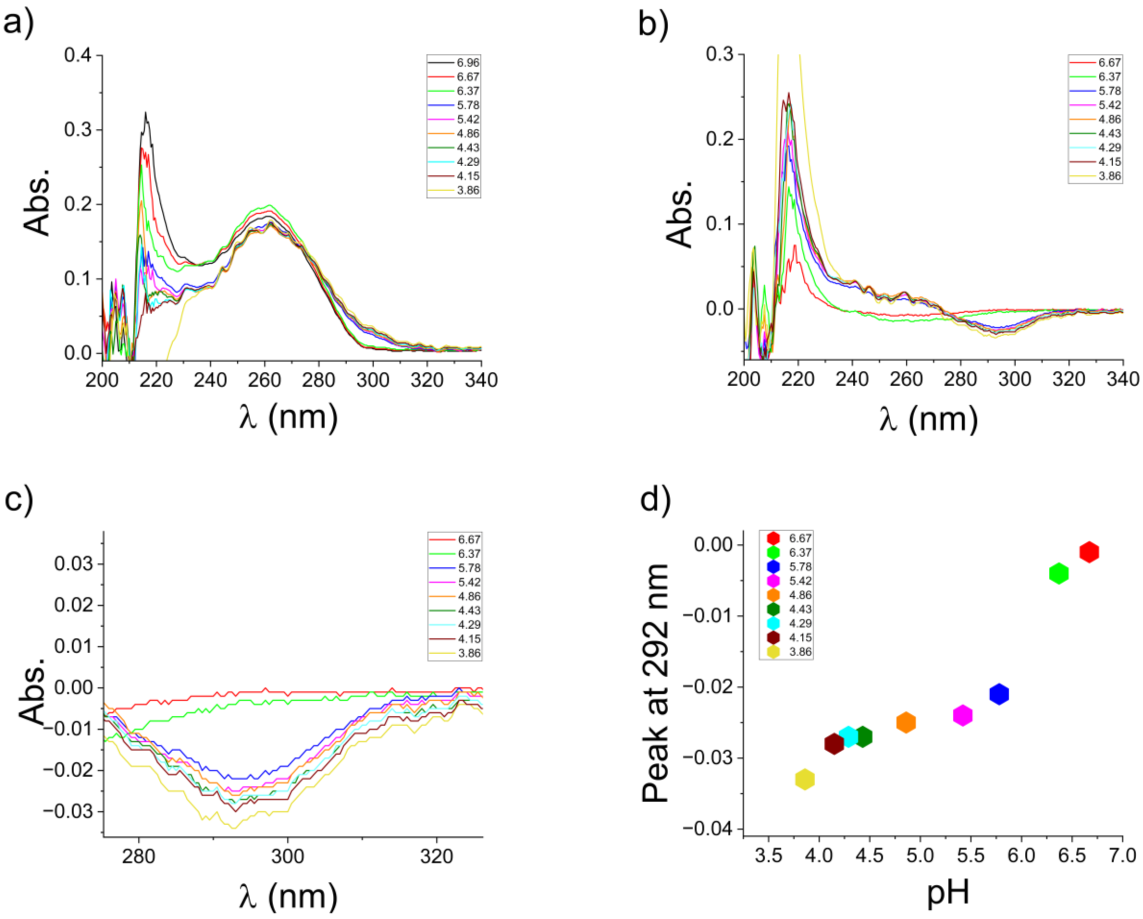


Fig. S1. (a) Representative original and (b) difference UV-vis spectra (UV-vis $_{pH=6.96}$ – UV-vis $_{low\ pH}$) for the DNA fragment, measured to establish i-motif formation propensity of the cytosine-rich strands in the low pH regime. (c-d) The progressive apparition of a minimum at ~292 nm with lowering the buffer pH, reveal characteristic of i-motif folds. At a lower pH (pH ~ 3.86) than that required for the stable i-motif formation, the bases of ssDNA become totally protonated, which alters the absorbance properties compared to neutral pH.

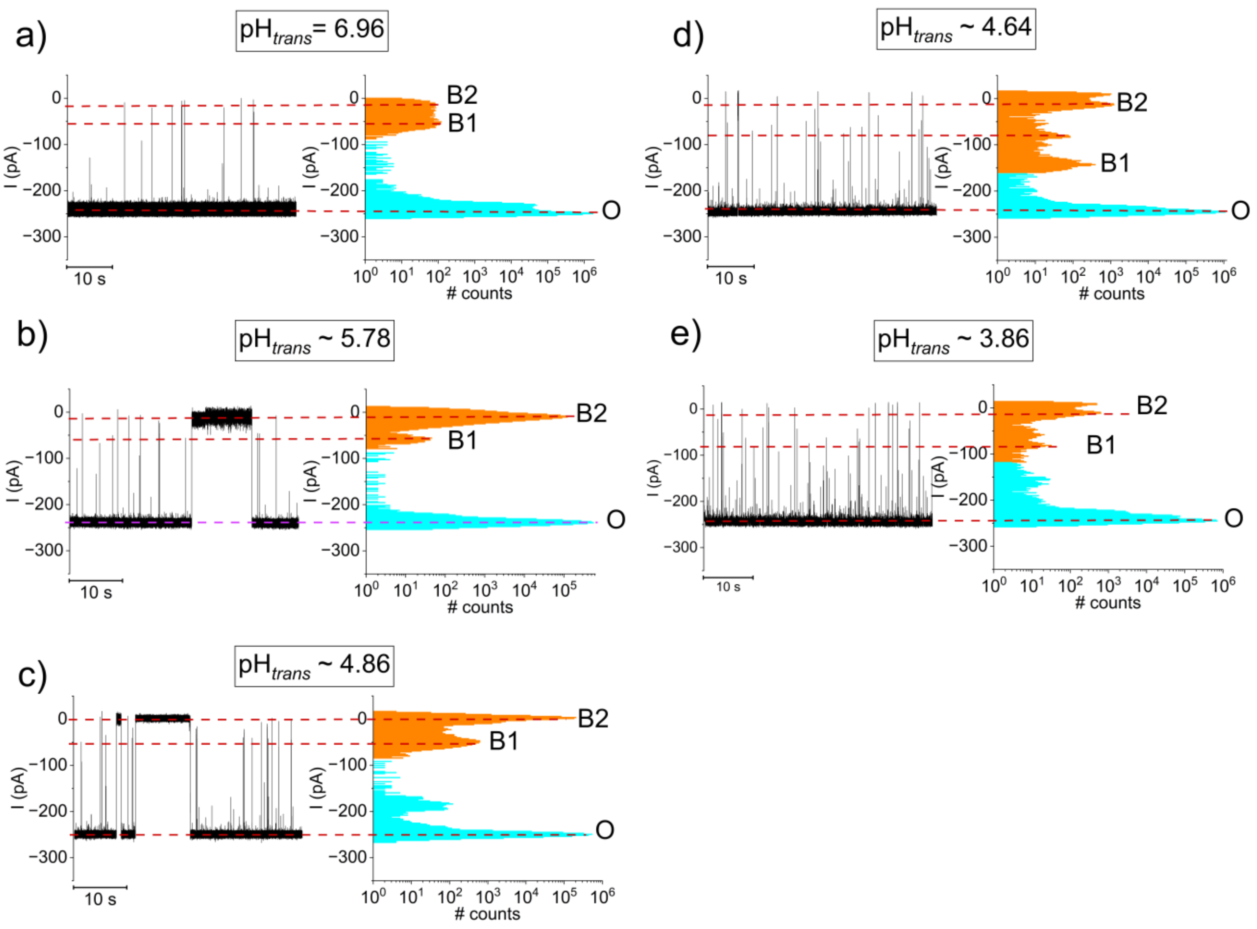


Fig. S2. **Detection of the i-motif at the β-barrel entrance of the α-HL.** Representative, single-channel current traces of 2 μM *trans*-added DNA capture and transport through the α-HL nanopore, recorded at ΔV = -100 mV in symmetrical 3 M KCl buffered with 10 mM HEPES at pH 6.96 (a) and around $pH_{trans}$ ~ 5.78 (b), ~ 4.86 (c), ~ 4.64 (d) and ~ 3.86 (e). Included for each representative trace are the corresponding all-point histograms, unraveling the ionic currents for the α-HL's open state (O), and the commonly found blockade levels induced by DNA-α-HL interactions, denoted by B1 and B2.

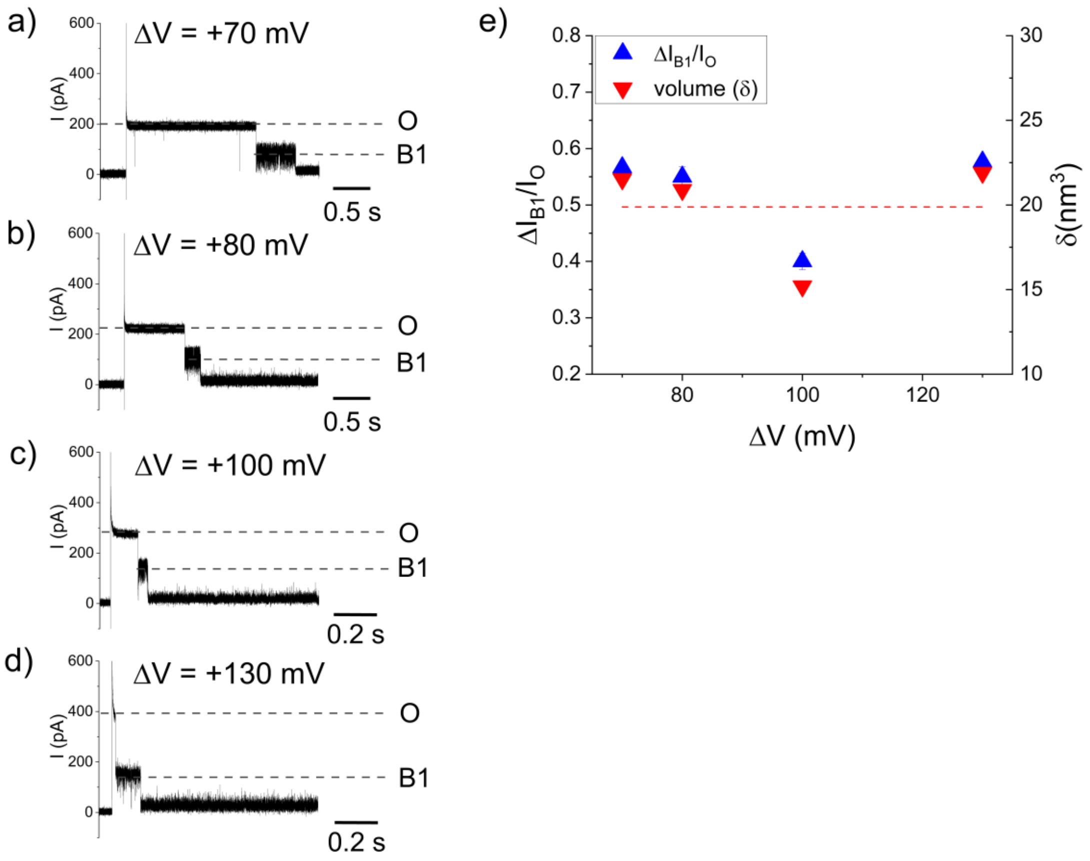


Fig. S3. **The irreversible capture of an i-motif inside α-HL's vestibule allows volumetric analysis in confined spaces**. (a-d) Representative traces illustrating the irreversible capture of *cis*-added DNA [2 μM] at with the *cis* electrolyte buffered at $pH_{cis}$ ~ 4.86, recorded at distinct ΔVs. The zoomed-in excerpts show representative blockade events triggered by the i-motif captured inside the nanopore, termed B1 and B2 (see also main text). (e) Voltage-dependence of the relative nanopore blockades ($\frac{\Delta I_{B1}}{I_O}$) elicited by a vestibule-trapped captured DNA (i-motif and the appended $dA_9$ extension) and the corresponding, calculated molecular volumes of the DNA (δ). The dashed line represents the zero-slope linear fit of the δ vs. ΔV plot.

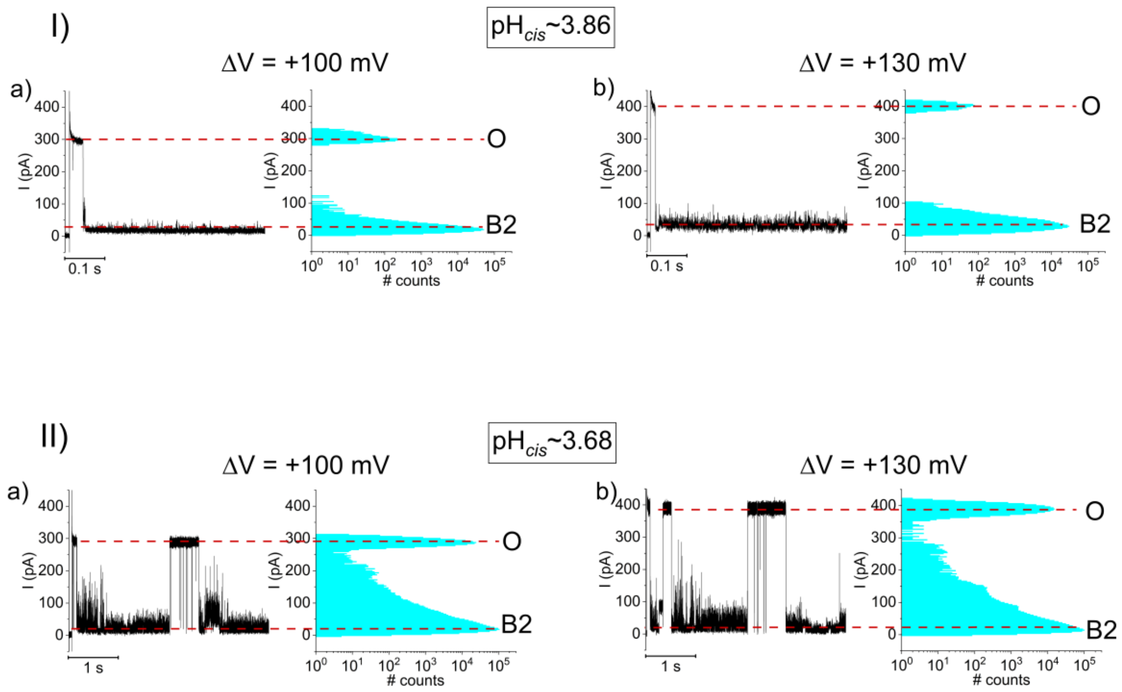


Fig. S4. **At low, acidic pHs, the vestibule-confined i-motif retains its stability**. Original traces with corresponding all-point histograms recorded through an α-HL nanopore showing ionic current blockades (state B2) induced by capture of *cis* added DNA [2 μM]. The measurements were performed in 3 M KCl buffered with 10 mM HEPES, with $pH_{cis}$ ~ 3.86 (I, a, b) or $pH_{cis}$ ~ 3.68 (II, a, b), at the indicated transmembrane potentials (ΔV). See also Table S3 for comprehensive statistical analysis on the ionic current blockades.

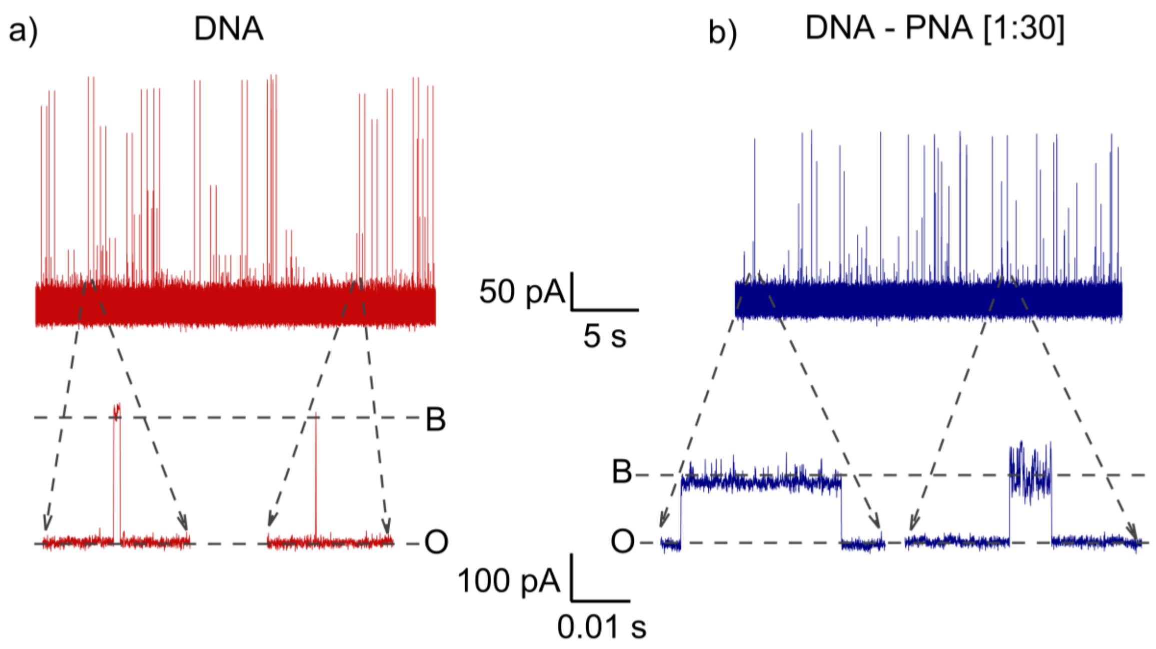


Fig. S5. **Complementary 6-mers PNA associate with DNA fragments and generate specific blockades when captured at the α-HL's β-barrel**. (a) Representative ionic current fluctuations induced across the α-HL nanopore upon DNA [1 μM] capture on the *trans* side at ΔV = -130 mV, 3M KCl, 10 mm HEPES and at neutral pH = 6.99. (b) Subsequent addition of 6-mer PNA [30 μM] to the *trans* side, generate DNA-PNA complexes whose capture at the α-HL's β-barrel entrance generate ~ one order of magnitude longer dissociation events ($\tau_{off}$) corresponding to the blocked state (B), as seen also the zoomed-in excerpts of the corresponding current traces displayed beneath ($\tau_{off}$(DNA) (s) = $0.002 \pm 6 \times 10^{-4}$ and ($\tau_{off}$(DNA-PNA) (s) = $0.01 \pm 0.002$) .

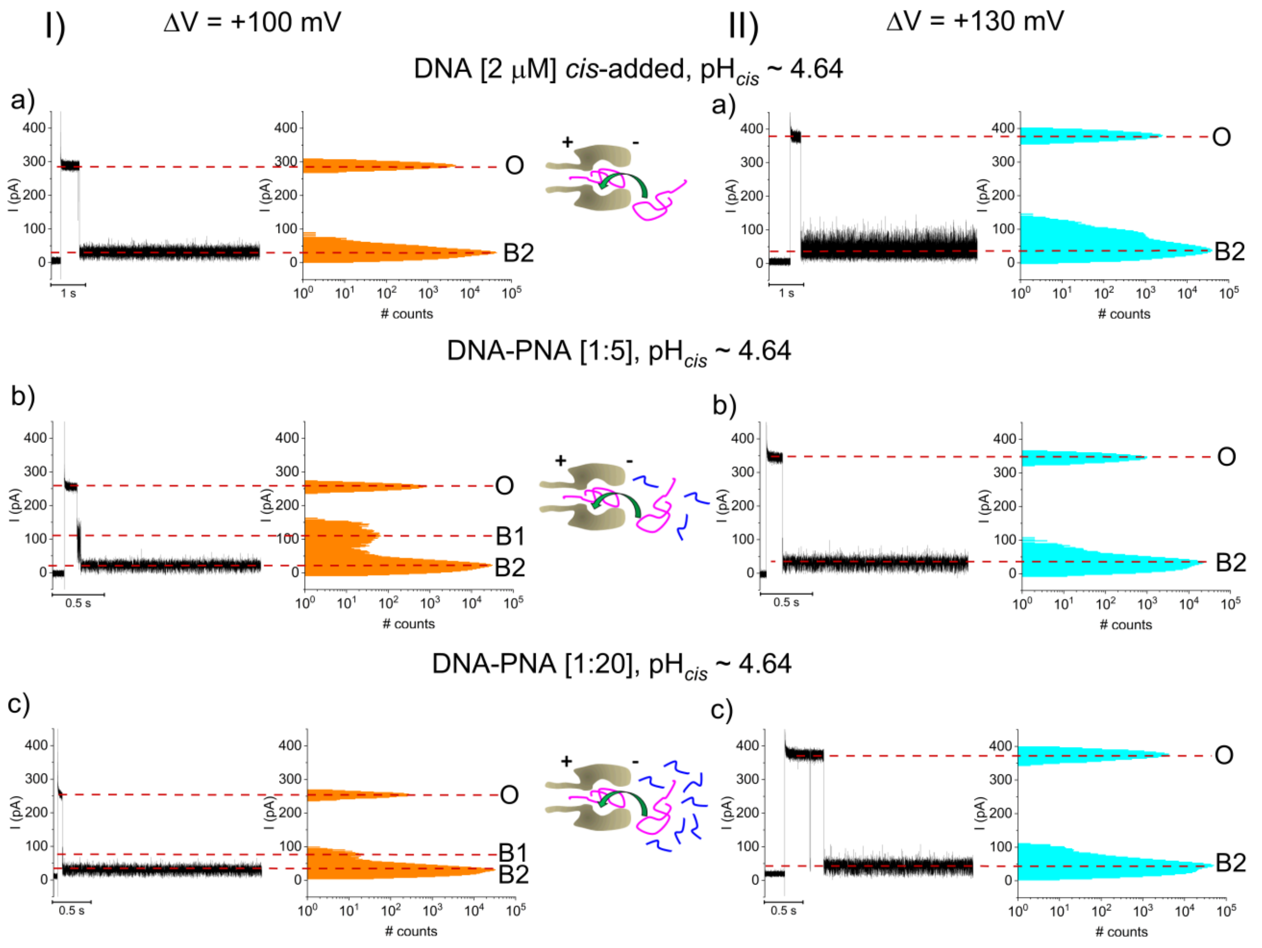


Fig. S6. **DNA-PNA interactions fail to interfer with the low pH induced i-motif formation if acidity change preceds 6-mers PNA addition.** Single-molecule recordings monitoring stochastic events illustrative of *cis*-added DNA-α-HL reversible interactions at $pH_{cis}$ ~ 4.64 recorded at ΔV = +100 mV (I, a-c ) and ΔV = +130 mV (II, a-c) in the absence or presence of PNA at distinct molar ratios, as indicated in the corresponding panels. The i-motif signature on the DNA is indicated by the prevalence of blockade substates B1 and B2 (see main text, also). As presented, 6-mers complementary PNA addition on the *cis* side, leaves the i-motif appearance largely invariant. The shown sketches illustrate DNA and respectively of DNA(magenta)-PNA(blue) fragments irreversible capture at α-HL's vestibule, under the specificed conditions. The comprehensive statistical analysis on the ionic current blockades is presented in Table S4.

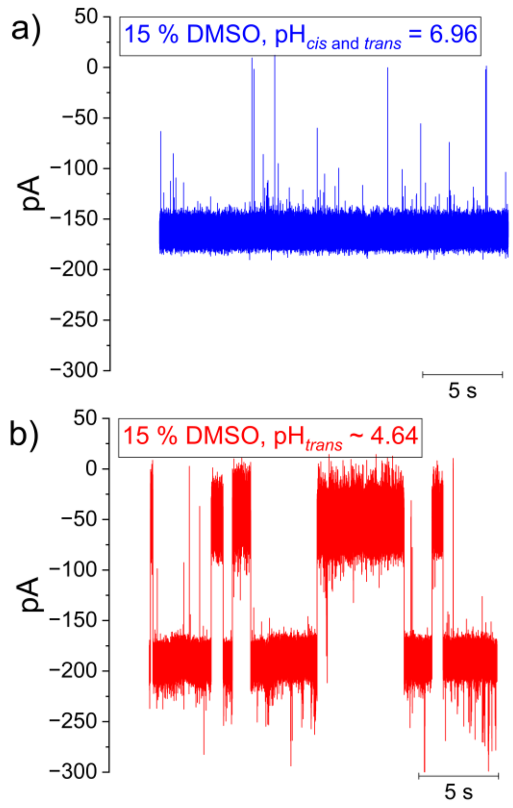


Fig S7. **DMSO does not preclude the low pH-induced i-motif formation.** (a) Representative traces illustrating *trans*-added DNA-α-HL reversible interactions recorded at ΔV = -130 mV in symmetrical 3 M KCl buffered with 10 mM HEPES at pH 6.96, and *trans*-added 15 % (v/v) DMSO. (b) By lowering the *trans*-side pH to ~ 4.64, longer lasting blockade events emerged, consistent with the discovery that such acidic $pH_{trans}$ values facilitate the formation of folded i-motif DNA species, likely to plug for longer times the nanopore upon capture.

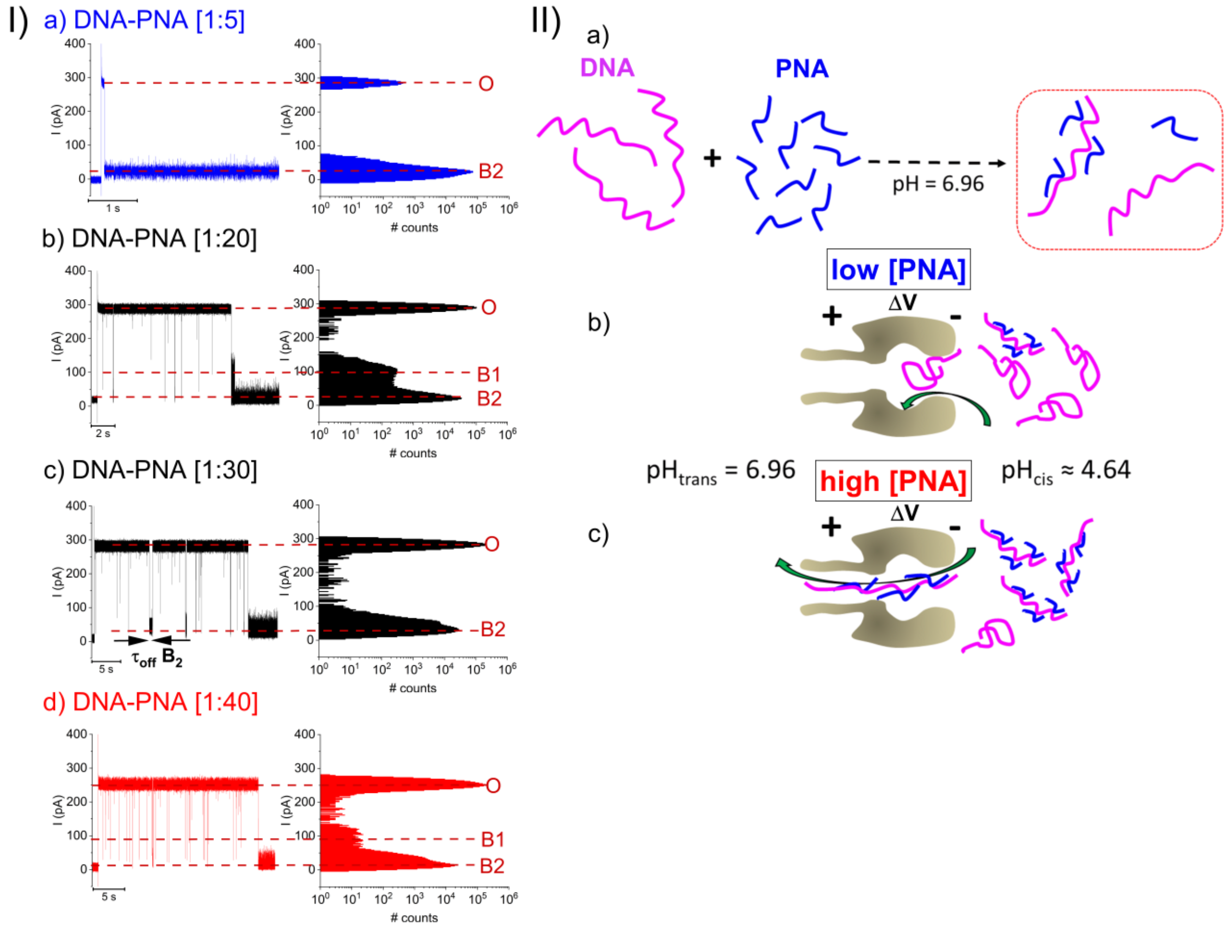


Fig. S8. **DNA-PNA hybridization at neutral pH and optimal molar ratio impede DNA folding into an i-motif structure, at subsequent low pH.** (I) Single-molecule recordings monitoring stochastic events illustrative of pre-incubated DNA-PNA fragments at neutral pH and different molar ratios of [1:5] (a), [1:20] (b), [1:30] (c) and [1:40] (d), captured from the *cis* chamber of the measuring setup containing an electrolyte with the $pH_{cis} \sim 4.64$ at $\Delta V = +100$ mV. By $\tau_{off}$ B2 we denote a representative duration of reversible B2 events, seen especially at high [PNA] vs. [DNA] (i.e., panels b-d), described also in Table S6 where we present the comprehensive statistical analysis on the ionic current blockades. (II) (a) Sketched view of DNA (magenta) and PNA (blue) hybridization in neutral pH conditions, generating DNA-PNA complexes with a distinct free equilibrium concentration [DNA-PNA], depending upon the DNA-PNA molar ratio. (b)

Subsequently, at $pH_{cis}$ ~ 4.64, addition of a mixture containing a low [PNA] vs. [DNA] resulted in a correspondingly large concentration of free DNA fragments which at the working acidic $pH_{cis}$ folded into an i-motif, blocking the $\alpha$-HL irreversibly upon capture on the *cis* side (see also I, a, in blue). (c) At $pH_{cis}$ ~ 4.64, addition of a mixture containing a high [PNA] vs. [DNA] resulted in a correspondingly large concentration of free DNA-PNA fragments which at the working acidic $pH_{cis}$ remained largely unfolded, trafficking the $\alpha$-HL upon capture on the *cis* side (see also, I, d, in red).
